# Genetically-barcoded SIV facilitates enumeration of rebound variants and estimation of reactivation rates in nonhuman primates following interruption of suppressive antiretroviral therapy

**DOI:** 10.1371/journal.ppat.1006359

**Published:** 2017-05-04

**Authors:** Christine M. Fennessey, Mykola Pinkevych, Taina T. Immonen, Arnold Reynaldi, Vanessa Venturi, Priyanka Nadella, Carolyn Reid, Laura Newman, Leslie Lipkey, Kelli Oswald, William J. Bosche, Matthew T. Trivett, Claes Ohlen, David E. Ott, Jacob D. Estes, Gregory Q. Del Prete, Jeffrey D. Lifson, Miles P. Davenport, Brandon F. Keele

**Affiliations:** 1 AIDS and Cancer Virus Program, Leidos Biomedical Research Inc., Frederick National Laboratory for Cancer Research, Frederick, Maryland, United States of America; 2 Infection Analytics Program, Kirby Institute for Infection and Immunity, UNSW Australia, Sydney, NSW, Australia; University of Wisconsin, UNITED STATES

## Abstract

HIV and SIV infection dynamics are commonly investigated by measuring plasma viral loads. However, this total viral load value represents the sum of many individual infection events, which are difficult to independently track using conventional sequencing approaches. To overcome this challenge, we generated a genetically tagged virus stock (SIVmac239M) with a 34-base genetic barcode inserted between the *vpx* and *vpr* accessory genes of the infectious molecular clone SIVmac239. Next-generation sequencing of the virus stock identified at least 9,336 individual barcodes, or clonotypes, with an average genetic distance of 7 bases between any two barcodes. *In vitro* infection of rhesus CD4+ T cells and *in vivo* infection of rhesus macaques revealed levels of viral replication of SIVmac239M comparable to parental SIVmac239. After intravenous inoculation of 2.2x10^5^ infectious units of SIVmac239M, an average of 1,247 barcodes were identified during acute infection in 26 infected rhesus macaques. Of the barcodes identified in the stock, at least 85.6% actively replicated in at least one animal, and on average each barcode was found in 5 monkeys. Four infected animals were treated with combination antiretroviral therapy (cART) for 82 days starting on day 6 post-infection (study 1). Plasma viremia was reduced from >10^6^ to <15 vRNA copies/mL by the time treatment was interrupted. Virus rapidly rebounded following treatment interruption and between 87 and 136 distinct clonotypes were detected in plasma at peak rebound viremia. This study confirmed that SIVmac239M viremia could be successfully curtailed with cART, and that upon cART discontinuation, rebounding viral variants could be identified and quantified. An additional 6 animals infected with SIVmac239M were treated with cART beginning on day 4 post-infection for 305, 374, or 482 days (study 2). Upon treatment interruption, between 4 and 8 distinct viral clonotypes were detected in each animal at peak rebound viremia. The relative proportions of the rebounding viral clonotypes, spanning a range of 5 logs, were largely preserved over time for each animal. The viral growth rate during recrudescence and the relative abundance of each rebounding clonotype were used to estimate the average frequency of reactivation per animal. Using these parameters, reactivation frequencies were calculated and ranged from 0.33–0.70 events per day, likely representing reactivation from long-lived latently infected cells. The use of SIVmac239M therefore provides a powerful tool to investigate SIV latency and the frequency of viral reactivation after treatment interruption.

## Author summary

Elucidation of HIV dynamics is essential for a thorough understanding of viral transmission, therapeutic interventions, pathogenesis, and immune evasion. The complex dynamics of reservoir establishment and viral recrudescence upon therapy removal present the primary obstacles to developing a functional cure. We sought to develop a virus model system for use in nonhuman primates that allows for the genetic discrimination of nearly 10,000 otherwise isogenic clones. This “synthetic swarm” adds a genetic component to viral dynamics where individual viral lineages can be tracked and monitored during infection. Here we utilized this model to identify the dynamics of viral reservoir establishment and rebound. We found that after 300 or more days of therapy, between 4 and 8 distinct viral lineages could be detected upon therapeutic intervention. Using the relative proportion of each distinct genetic barcoded virus and the overall viral load curve, we could estimate the time and rate of reactivation from latency. On average, we found 1 reactivation event every 2 days with reactivation of the first rebounding variant within days of therapeutic interruption. This virus model will be useful for testing various approaches to reduce the latent viral reservoir and to molecularly track viral dynamics in all stages of infection.

## Introduction

A major obstacle to developing a cure for HIV is the establishment in early infection of long-lived viral reservoirs, defined as sources of virus that can persist over extended periods despite seemingly effective suppressive combination antiretroviral therapy (cART), that can cause recrudescent viremia if cART is interrupted. While multiple anatomic sites and cell compartments likely act as viral reservoirs, it has been argued that latently infected resting CD4+ T cells represent the most significant long-lived viral reservoir for HIV-1 [1–7]. During latency, these reservoirs are unrecognized by host immune responses and cells containing integrated latent proviruses are unaffected by current cART, which acts only by blocking new rounds of infection. For patients to safely stop treatment, the immune system must be able to control rebound infection (sustained cART free remission or functional cure), or all reactivatable replication-competent virus must be completely eradicated.

Numerous studies are in progress to test therapies designed to decrease viral reservoir size and prolong ART-free remission. A critical element for evaluating the effectiveness of these therapies is an accurate measurement of reservoir size before and after treatment. These assessments have typically involved *ex vivo* estimates and have been based on total cell-associated viral DNA (CA-vDNA) measurements [8–10], stimulation of PBMCs or enriched CD4+ T cells to measure the frequency of cells producing viral RNA (vRNA induction assay or TILDA) [11–14] or the frequency of cells harboring replication competent virus (quantitative viral outgrowth assays, QVOA)[1, 3, 13, 15, 16]. However, each method for estimating reservoir size has shortcomings. Ho et al. demonstrated that the QVOA tends to underestimate the amount of replication competent virus present in any sample, as not all latent proviruses will reactivate after a single stimulation event [16]. Additionally, QVOA requires large source specimens, is time and labor intensive, and has limited precision and dynamic range. On the other hand, PCR detection of viral DNA tends to greatly overestimate the reservoir size, as much of the viral DNA detected in these assays does not encode full length replication competent virus due to large deletions or APOBEC mediated mutations. While the vast majority of intact, APOBEC mutation-free genomes are replication competent and could contribute to rebound viremia [16] identifying and quantifying these genomes requires near-full genome sequencing which is time consuming and expensive, necessarily precluding its use in large cohorts of patients. While accurate assessment of the size of the viral reservoir is central to the evaluation of HIV cure strategies, none of the *ex vivo* assays directly assess the size of the viral reservoir that can lead to recrudescent viremia after cART interruption.

Most studies evaluating the effects of novel therapies on viral reservoir size are dependent on these *ex vivo* assays, however due to sample size and assay sensitivity issues, “undetectable” viral measurements do not necessarily indicate an absence of reactivatable virus, so as experimentation progresses, ultimately these treatments still require testing in HIV+ patients with the eventual discontinuation of cART to test for functional cure. In these instances, time to rebound after treatment interruption is considered the most direct measure of cure intervention treatment efficacy [17]. This approach might be effective for revealing large differences between treatment groups, which cause significant differences in time to detectable rebound viremia, however effects of treatments that result in small but potentially meaningful changes in reservoir size may be too subtle to be detected with this approach [17]. This will be particularly true for individuals with large reservoirs where even large differences between treatment groups will be difficult to detect using only time to rebound, and in groups with highly divergent interpatient reservoir size which will affect time to detectable rebound viremia. Therefore, alternative approaches for evaluating the functional reservoir size (i.e. the cells that can contribute to systemic viremia once therapy is removed) and the effects of new therapeutic interventions on the reservoir size are needed.

AIDS virus infected non-human primates (NHPs) represent useful models to study viral reservoir establishment and to evaluate changes in reservoir size with novel interventions. Until recently, consistent and complete viral suppression was difficult to achieve in SIV-infected rhesus macaques with cART regimens developed for HIV-1 infection in humans. However, there are now several classes of drugs, including nucleos(t)ide reverse-transcriptase inhibitors, protease inhibitors, and integrase inhibitors that have been evaluated and shown to be effective for suppression of SIV and SHIVs in infected macaques. Recently, cART regimens have been developed that can effectively, durably and sustainably reduce plasma vRNA to clinically relevant levels (below 15–50 copies per mL) [18–22]. These regimens result in similar viral suppression dynamics to those observed in humans. Additionally, drugs are typically administered daily without any “drug holidays” or accidental missed doses. Frequent blood sampling and standardized assays provide assurances of successful suppression. Finally, NHPs may be removed from cART without the ethical implications involved in removing HIV-1 infected humans from treatment.

To more fully realize the potential of NHP models for evaluation of candidate cure approaches, we developed a novel, barcoded virus system that allows for a deep genetic assessment of the number of rebounding viruses, in conjunction with time to rebound viremia measurements. This novel barcoded virus is fully and stably replication competent *in vitro* and *in vivo* and can be used to establish infection with a large number of otherwise sequence identical viral clonotypes bearing unique barcode sequences. Following cART treatment and interruption, the number and relative proportion of each rebounding clonotype can be measured with next generation sequencing of the barcodes, using high template input that allows for the discrimination of individual rebounding clonotypes. By combining viral growth rates (the rate at which the virus grows once achieving detectable systemic infection) and the relative proportion of each rebounding clonotype, the frequency of rebound of each clonotype can be estimated in each animal. This approach is likely more sensitive than measuring time to detectable viremia alone because it is less affected by natural variation among individual animals, and consequently requires smaller group sizes to distinguish statistically significant differences in reservoir size. This approach allows for detection of both small or large changes in the viral reservoir population, a distinction which may be critical for evaluating interventions resulting in real, but only modest changes in reservoir size. Our use of this system in initial *in vivo* studies demonstrates that the time of initiation and duration of cART administration in NHPs can alter the size of the reservoir, allowing for tightly controlled experimental design and execution, an idea also introduced by Whitney et al (19). These data will help inform HIV cure research by providing a basic understanding of the biology of latency establishment, maintenance, and reactivation and will facilitate evaluation of potential therapies intended to reduce reservoir size.

## Methods

### Ethics statement

Twenty-six purpose-bred Indian-origin male rhesus macaques (*Macaca mulatta*) weighing on average 7kg (range 5-9kg) were housed at the National Institutes of Health (NIH) and cared for in accordance with the Association for the Assessment and Accreditation of Laboratory Animal Care (AAALAC) standards in an AAALAC-accredited facility and all procedures were performed according to protocols approved by the Institutional Animal Care and Use Committee of the National Cancer Institute (Assurance #A4149-01). Animals were maintained in Animal Biosafety Level 2 housing with a 12:12-hour light:dark cycle, relative humidity 30% to 70%, temperature of 23 to 26°C and all animals were observed twice daily by the veterinary staff. Filtered drinking water was available ad libitum, and a standard commercially formulated nonhuman primate diet was provided thrice daily and supplemented 3–5 times weekly with fresh fruit and/or forage material as part of the environmental enrichment program. Environmental enrichment: Each cage contained a perch, two portable enrichment toys, one hanging toy, and a rotation of additional items (including stainless steel rattles, mirrors, and challenger balls). Additionally, the animals were able to listen to radios during the light phase of their day and were provided with the opportunity to watch full-length movies at least three times weekly. At the start of the study, all animals were free of cercopithecine herpesvirus 1, simian immunodeficiency virus (SIV), simian type-D retrovirus, and simian T-lymphotropic virus type 1. All animals were treated with enrofloxacin (10 mg/kg once daily for 10 days), paromomycin (25 mg/kg twice daily for 10 days), and fenbendazole (50 mg/kg once daily for 5 days) followed by weekly fecal culture and parasite exams for 3 weeks to ensure they were free of common enteric pathogens. At least a 4-week post-treatment period allowed time for stabilization of the microbiome prior to use in this study.

### Construction of SIVmac239M

Primers were designed to introduce an MluI restriction site between the *vpx* and *vpr* accessory genes. These primers contain regions complementary to either the *vpx* or *vpr* genes with the MluI restriction site appended to the 3’ end of each primer. Amplicons were generated from SIVmac239 template using a generic primer upstream of SbfI and downstream of the EcoRI restriction site. The *vpx*-containing fragment was digested with MluI and SbfI, and the *vpr*-containing fragment was digested with MluI and EcoRI. SIVmac239 plasmid digested with SbfI and EcoRI was ligated with the two digested amplicons overnight at 16°C using T4 DNA ligase (NEB). 5μL of the ligation reaction was transformed into Stbl2 cells (Invitrogen) and plated on agar plates containing 100μg/mL ampicillin. Resulting colonies were checked for correct assembly and insertion of the MluI site. This clone was termed SIVmac239-Mlu.

**Fig 1:**
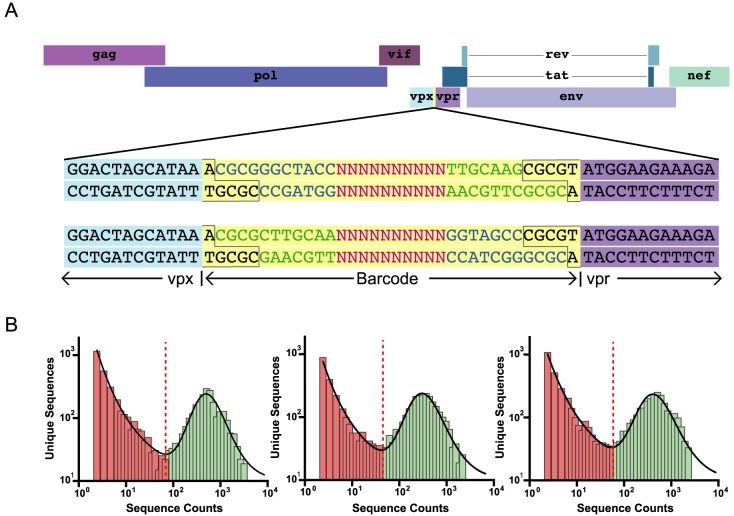
Insertion of genetic barcode into SIVmac239. (A) A 34 base cassette (yellow) bearing a stretch of 10 random bases was inserted between *vpx* and *vpr* of wild-type SIVmac239 to generate the genetically barcoded virus SIVmac239M. The MluI restriction site used is outlined in black, and the sequences of the barcode flanking regions are colored in blue and green to depict the two possible insertion orientations. (B) Representative sequences in single stock aliquots depicting the bimodal distribution of authentic barcodes (green) versus barcodes containing PCR error (red).

The barcode insert was synthesized as single stranded forward and reverse barcoded templates (IDT) that were comprised of 10 random bases, flanked on either end by a stretch of bases complementary to the same region on the opposite primer to function as a molecular “clamp” with MluI sticky ends on both ends of the dimer (Fig 1A). To generate primer dimers, the forward and reverse barcode primers were mixed in equal proportion and heated to 95°C. The temperature was slowly lowered at a rate of 1.5°C/min to allow primer pairs to anneal.

SIVmac239-Mlu was digested with MluI and the DNA was purified with a Qiaex II kit. The digested SIVmac239-Mlu and primer dimers were mixed and ligated at 16°C overnight. The primer sequence was designed such that upon ligation of the primer into the MluI site of the SIVmac239-Mlu, the MluI site would be destroyed. Thus, the ligation product was digested again with MluI to linearize any genome not containing a primer dimer insert, and the digestion product cleaned and purified with a Qiaex II kit. The eluted product was transformed into Stbl2 cells, and transformants were grown up in LB amp overnight. The plasmid library was extracted from the bacterial preparations using the Qiagen MaxiPrep kit.

### SIVmac239M viral stocks

Virus was prepared in HEK-293T cells transfected with the prepared SIVmac239M plasmid library using Mirus Trans-IT 293 transfection reagent as described by the manufacturer. Culture medium was changed at 24hr post-transfection, and culture supernatants were collected at 48hr. Supernatants were passed through a 0.45μm filter and stored at −80°C in 0.5 or 1 mL aliquots. Viral infectivity was determined using TZM-bl reporter cells (reference no. 8129; NIH AIDS Research and Reference Reagent Program), which contain a Tat-inducible luciferase and β-galactosidase gene expression cassette. Infectivity was determined by assessing the number of β-galactosidase expressing cells present after infection with serial dilutions of viral stocks. After dilution correction, wells containing blue cell counts falling within a linear range were averaged and used to determine the titer of infectious units (IU) per mL in the viral stock as previously described [23].

### MiSeq sample preparation

RNA was isolated from plasma or viral stock using QIAamp Viral RNA mini kit per manufacturer’s instructions. RNA was eluted from the column with 65μL elution buffer. cDNA was synthesized from the extracted DNA using Superscript III reverse transcriptase (Invitrogen) and a reverse primer (Vpr.cDNA3: 5’-CAG GTT GGC CGA TTC TGG AGT GGA TGC-3’ at position 6406–6380). The reaction mixture was prepared as previously described with initial incubation at 50°C for one hour then increased to 55°C for an additional hour. Temperature was increased to 70°C, and the reaction incubated for 15 minutes. Each reaction was then treated with RNaseH and incubated at 37°C for 20 minutes. qRT-PCR was used to quantify the cDNA synthesized in the previous step using the primers VpxF1 5’-CTA GGG GAA GGA CAT GGG GCA GG-3’ at 6082–6101 and VprR1 5’-CCA GAA CCT CCA CTA CCC ATT CATC-3’ at 6220–6199.

PCR was used to amplify the cDNA and add MiSeq adaptors directly onto the amplicon. Reactions were prepared using High Fidelity Platinum Taq per the manufacturer’s instructions, using primer VpxF1 and VprR1 combined with either the F5 or F7 Illumina adaptor sequence containing a unique 8 nucleotide index sequences. Template input values ranged from 5x10^3^ copies to 1x10^6^ copies. Reaction conditions used are as follows: 94°C, 2min; 40x [94°C, 15sec; 60°C, 1:30min; 68°C, 30sec]; 68°C, 5min.

Following PCR, 10μL from each reaction was pooled and purified using the QIAquick PCR purification kit. The resulting eluted DNA was quantified using the QuBit. The combined DNA sample was diluted to 3.0nM and 5μL of this diluted sample was placed in a new tube and denatured with 5μL 0.2N NaOH. This sample was vortexed and centrifuged at 280x*g* for 1 minute. The sample incubated at room temperature for 5 minutes, and 990μL of chilled HT1 buffer added. This sample was then diluted to 12.5pM.

The control PhiX library was treated similarly. 2μL of the PhiX library was combined with 3μL Tris-HCl pH 8.5, 0.1% tween-20. 5μL of 0.2N HCl was added to the library, and the sample vortexed and centrifuged at 280x*g* for 1 minute. The sample was incubated at room temperature for 5 minutes, and 990μL of chilled HT1 buffer added. Multiplexed samples and PhiX library were then loaded on the MiSeq reagent tray, and the run initiated.

### Single genome amplification

For low-template samples, we used single genome amplification (SGA) followed by direct Sanger sequencing to assess the frequency and number of unique barcodes. cDNA synthesis and PCR was performed as described above but using a limiting dilution of cDNA prior to PCR amplification. This method provides representative proportionality and excludes PCR-induces errors [24].

### Sequencing analysis

Samples that were multiplexed were separated into individual samples using Geneious software and the unique 8-nucleotide index. After barcode splitting, individual barcoded clonotypes were identified by sequencing the first 50 bases of *vpr*. The 34 bases immediately upstream of this alignment were extracted and presumed to encode the inserted barcode region. Sequences obtained from infected animals were identified by comparison to all barcodes identified in the stock (14,357). Only identical matches to a defined barcode were counted as an authentic input sequence. Given the short duration of infection prior to initiation of cART and the limited size of the insert, the vast majority of sequences were identical to a known barcode and were thus identified.

Some identified barcodes contained 1 or more deletions in the insert region. This deletion resulted in 1 or more bases of *vpx* necessarily included in the extracted “barcode”. Although these unique but shorter barcodes represent only 0.7% of all inserts observed, they were included in the comprehensive tally of all barcodes because they remain a unique and genetically identifiable insert.

Since all samples were quantified by real-time PCR, the theoretical limit of detection was estimated for each sample as the minimum number of sequences that would result from a single copy of an input template. Sequences below this threshold were discarded.

### *In vitro* analysis of barcoded virus

Viral replication curves were prepared by culturing CD8+ T-cell-depleted Indian-origin rhesus macaque peripheral blood mononuclear cells (PBMCs) (CD8+ depletion performed using Miltenyi Biotec CD8+ microbeads) in RPMI supplemented with 10% fetal bovine serum (FBS), 2mM l-glutamine, and 100U/mL penicillin and 100μg/mL streptomycin (RPMI-complete), stimulated for 3 days with 5μg/mL phytohemagglutinin (PHA) and IL-2 (100U/mL). Stimulated PBMC cultures were infected with SIVmac239 or SIVmac239M at an MOI of 0.01 or 0.001 (as determined by TZM-bl). 24hr post-inoculation, cell cultures were washed with phosphate buffered saline (PBS) twice and once with RPMI-complete to remove excess virus. Viral replication was monitored over 14 days by detection of supernatant SIV p27 antigen in an enzyme-linked immunosorbent assay (ABL) according to the manufacturer’s provided protocol.

### Animal study

In total, 26 animals were intravenously infected with 2.2x10^5^ IU (1mL) of transfection produced SIVmac239M. All 26 animals were used to enumerate the number of detectable barcodes measured during primary infection. Of these 26 animals, two animals were followed for over 3 months to assess early viral replication kinetics (peak and set point viral load) of SIVmac239M. Four of the other infected animals began antiretroviral treatment beginning at day 6 post infection and continued for 82 days. Each animal received a combination antiretroviral therapy (cART) regimen comprising a co-formulated preparation containing the reverse transcriptase inhibitors tenofovir (TFV: (R)-9-(2-phosphonylmethoxypropyl) adenine (PMPA), 20 mg/kg) and emtricitabine (FTC; 50 mg/kg) administered by once-daily subcutaneous injection, plus raltegravir (RAL; 150-200mg) given orally twice daily. At the time of interruption from cART, three animals were infused at the day of cART interruption with autologous CD8+ T cells transduced with an anti-SIV Gag T-cell receptor (animals MK9 and KTM) or with an irrelevant receptor (animals KMB and KZ2) plus daily subcutaneous injections of IL-2 at 10,000 IU/kg for 10 days. The total number of infused cells ranged from 4.6 to 6.4x10^9^ cells with <1% of the cells CD4+. In these animals, infused cells did not traffic to lymphoid or GI tissues and persistence of the cells was poor. Animal KTM died due to procedural complications at the time of cART interruption and was therefore excluded from subsequent analyses. In a separate cohort of 6 animals (study 2), therapy was initiated on day 4 post-infection with the same therapeutic regimen (TFV, FTC, RAL) described above with the addition of the protease inhibitor indinavir (IDV; 120mg BID) and ritonavir (RTV; 100mg BID) for the first 9 months. In study 2, cART treatment was continued for 305, 374, or 482 days, with two animals discontinuing therapy at each time point. The remaining 14 animals were used to enumerate the number of replicating clonotypes during primary infection.

### *In vivo* sample collection

Whole blood was collected from sedated animals. Plasma for viral RNA quantification and PBMCs for proviral DNA assays were prepared from blood collected in EDTA Vacutainer tubes (BD). Following separation from whole blood by centrifugation, plasma aliquots were stored at 80°C. PBMCs were isolated from whole blood by Ficoll-Paque Plus (GE Healthcare) gradient centrifugation.

### Plasma viral load determination

Plasma viral load determinations for SIV RNA were performed over the duration of the study using quantitative real-time PCR as described previously [25]. The limit of detection of this assay is 15 vRNA copies/mL.

### Quantitative evaluation of cell-associated DNA and RNA

Quantitative assessment of cell-associated viral DNA and RNA in PBMC pellets was determined by the hybrid real-time/digital RT-PCR and PCR assays essentially as described in Hansen et al. [26] but specifically modified to accommodate cell pellets. 100μL of TriReagent (Molecular Research Center, Inc) was added to cell pellets in standard 1.7mL microcentrifuge tubes and the tubes sonicated in a Branson cup horn sonicator (Emerson Electric, St. Louis) for 15 seconds at 60% amplitude to disrupt the pellet. Additional TriReagent was added to a final volume of 1mL and the remainder of the protocol was carried out as described previously [26]. Limit of detection is evaluated on a sample by sample basis, dependent on the number of diploid genome equivalents of extracted DNA assayed.

### Mathematical model of distribution of sequences in each stock aliquot

SIVmac239M viral stock was randomly distributed into 168 aliquots with 5,000 viral cDNA templates per aliquot. After next-generation sequencing of the barcode region, a bimodal frequency distribution of the number of copies of a given sequence in a single aliquot was observed. Many sequences were present at very low copy number, likely representing erroneous sequences generated during the PCR amplification and/or sequencing process. By contrast, sequences present at high copy numbers (representing authentic ‘input’ barcode sequences) were also observed in each aliquot. A mixture model approach was used to model the frequency of both the erroneous and input sequences. If *X* is a random variable corresponding to the number of copies of an individual sequence, then the distribution of *X* in an aliquot *f*(*x*) can be modeled as a mixture distribution of *X* for the erroneous sequences *f*_*E*_(*x*) and the authentic barcode input sequences *f*_*I*_(*x*). This can be written as:

$$f(x)=pf_{E}(x)+(1-p{)f}_{I}(x)$$ (1)

where *p* is the proportion of erroneous sequences in an aliquot, and (1−*p*) is the proportion of input sequences in an aliquot. Based on observed sequences, we fitted a model where the number of copies of the input sequences follows a lognormal distribution, while the erroneous sequences follow a power law distribution. The above distribution function is fitted to the number of copies of each unique sequence in an aliquot, using the function *mle* from MATLAB (R2014b). An optimal cutoff number of copies of a sequence for each aliquot was determined as the value where the theoretical distribution in the mixture model reaches a minimum. The sequences above the cutoff were designated putative input sequences and the sequences below the cutoff putative erroneous sequences. Moreover, the percentage of input sequences classified as erroneous and the number of erroneous sequences classified as input was estimated.

### Mathematical model to describe the distribution of sequences across 168 aliquots

The method above identifies the number of putative input sequences in each aliquot, however we also estimate that 2–5% of these are actually erroneous sequences that are classified as input barcodes. Since generation of PCR/sequencing error is likely a random event in a given aliquot, we might expect that most erroneous sequences will be confined to one or a few of the 168 aliquots. However, since we expect ≈10,000 total input sequences in the stock, and observe around 2000 sequences per aliquot, then we should see most input sequences in many aliquots. Based on this observation, the probability of observing a sequence in *n* aliquots is given by the mixture of binomial distributions:

$$p(n)=fBin(N,p_{1})+(1-f)Bin(N,p_{2})$$ (2)

in which *p*_1_ is the probability of observing the erroneous sequences, *p*_2_ is the probability of observing an input sequence, and *f* is the proportion of erroneous sequences. The above distribution function is fitted to the histogram of the number of aliquots each sequence is observed in (using the function *mle* in MATLAB v. R2014b). Using the fitted distribution function, we could find a cutoff value that can be used to determine the total number of input sequences across all aliquots. We can also estimate the false positive and false negative rates around this cutoff. Additionally, we also tested for a binomial model with non-constant proportion in the input sequences. However, allowing for a distribution in the proportion of input sequences did not yield a better fit (p = 0.78, likelihood ratio test), hence we found no evidence for a distribution in clone size of the input sequences.

### Mathematical model for estimating reactivation frequencies

In order to estimate frequency of reactivations, we assumed exponential viral growth at the earliest stage of infection.

$$V=V_{0}e^{g(t-t_{0})}$$ (3)

The time between *i*^*th*^ and (*i* + 1)^*th*^ reactivations, Δ_*i*_ = *t*_*i*+1_ − *t*_*i*_, can be estimated from ratios $R_{i}=\frac{V_{i}}{V_{i+1}}=e^{g(t_{i+1}-t_{i})}$, *i* = 1,…,*n* − 1 of rebounders as shown by the following formula:

$$\Delta_{i}=\frac{\ln\;R_{i}}{g}.$$ (4)

In order to find the growth rate *g* of each rebounder (assumed to be the same), we assume that reactivation occurs in average every Δ days. Thus the total viral load (i.e.: the sum of all variants) at time *t* after treatment can be expressed by formula:

$$V(t)=V_{0}e^{g(t-t_{0})}+V_{0}e^{g(t-t_{0}-\Delta )}+V_{0}e^{g(t-t_{0}-2\Delta )}\ldots +V_{0}e^{g(t-t_{0}-(n-1)\Delta )},(n-1)=\lfloor (t-t_{0})/\Delta \rfloor .$$ (5)

Taking into account that (*e*^−*g*Δ^)^*m*^, *m* = 0,…,*n* − 1, is a geometric progression, we can reduce the function (5) so it will take the form:

$$V(t)=V_{0}e^{g(t-t_{0})}\frac{1-e^{-g\Delta (\lfloor \frac{t-t_{0}}{\Delta }\rfloor +1)}}{1-e^{-g\Delta }},$$ (6)

where ⌊*x*⌋ is the largest integer not greater than *x*.

The function (6) has discontinuity that may create some obstacle in finding the global minimum during fitting. Thus, for the purpose of fitting we removed the discontinuities in (6) by substituting ⌊(*t* − *t*_0_)/Δ⌋ with (*t* − *t*_0_)/Δ and rewrite the expression (6) for the log of viral load:

$$\ln\;V(t)=\ln\;V_{0}+\ln\left(e^{g(t-t_{0})}-e^{-g\Delta }\right)-\ln\left(1-e^{-g\Delta }\right)$$ (7)

In order to use average time between reactivations that can be obtained from the ratios of founder virus data, as it was described above, we substitute Δ in (7) by the estimate of the mean, $\bar{\Delta }=\frac{\bar{L}}{g}$, where $\bar{L}=\frac{1}{\left(n-1\right)}\sum_{i=1}^{n-1}\ln\;R_{i}$. Thus, we obtain the formula:

$$\ln\;V(t)=\ln\;V_{0}+\ln(e^{g(t-t_{0})}-e^{-\bar{L}})-\ln(1-e^{-\bar{L}}),$$ (8)

where *n* is the number of founder viruses in the dataset. Model was fitted (using Prism 6.07, GraphPad Software Inc. San Diego, Ca, USA) to exponential phase of growth of virus in monkeys having *V*_0_ as a shared parameter.

## Results

### Generation and characterization of a genetically barcoded virus stock

We reasoned that a molecularly barcoded SIV clone would have great utility for studies of HIV/SIV latency, viral reservoir establishment and maintenance, and viral rebound upon therapeutic interruption. To generate this barcoded virus, the MluI restriction recognition sequence (ACGCGT) was introduced into the SIVmac239 infectious molecular clone (IMC) between the stop codon of *vpx* and the start codon of *vpr*. A genetic cassette consisting of 10 random bases with 7 complementary bases flanking each end was ligated into the SIVmac239 clone using the introduced MluI restriction site (Fig 1A). Importantly, the genetic insert is bidirectional, effectively doubling the discriminating power of the barcode. Following ligation, a large bacterial plasmid library was generated and was then used for large-scale virus production via transfection of HEK-293T cells. All produced virus was collected, pooled, and aliquoted, such that single aliquots contain a representative sampling of all genetic variants generated. Thus, the generated virus stock contained variants of SIVmac239 that differed only within a 34-nucleotide insertion harboring a 10 base-stretch of random nucleotides in an otherwise genetically clonal genome. These 34 bases comprise the viral barcode and the virus stock was designated SIVmac239M.

The goal of generating SIVmac239M was to produce a phenotypically homogeneous viral population with extensive diversity contained entirely within a small region of the genome suitable for deep sequencing and with a known distribution of the genetically distinct viral barcodes (or viral clonotypes). Therefore, it was necessary to determine the genetic diversity and abundance of each clonotype in the virus stock. When sequencing such a large potential number of genetic variants, it can be difficult to discern between sequences arising from PCR or sequencing error and those representing true input viral clonotypes. To distinguish these sequences, viral RNA was extracted, synthesized into cDNA, and distributed into 168 aliquots each containing 5,000 viral templates. Following PCR and Illumina-based sequencing of each aliquot, the number of unique sequences was compared to the total sequence count. Using a limited template input with massive oversampling of sequencing (at least 100-fold over-sequencing per template), we found a clear bimodal distribution of both PCR-induced errors (power-law distributed, with a high proportion of single sequences), and authentic clones (log-normally distributed, with sequences present at high frequency) (Fig 1B). We then identified the threshold number of copies separating the erroneous from the authentic barcode sequences in each aliquot. Using this approach, we detected a total of 14,357 unique clonotypes across the 168 aliquots. These clonotypes were then rank ordered by the number of replicate aliquots in which each was found. Of the 5,021 sequences found in only one aliquot, we estimated that only approximately 100 of these were likely to be authentic input barcodes based on the distribution of the 168 aliquots (Eq 2). Therefore, the vast majority of input barcodes were contained within the top 9,336 sequences.

Phylogenetic analysis of these 9,336 identified clonotypes was performed to determine the genetic relatedness of each barcoded clone (S1 Fig). Of the 9,336 identified barcodes, 5,519 were inserted in one direction, and 3,817 were inserted in the inverse direction. To quantify genetic relatedness between barcodes, we performed pairwise comparisons of each barcode (S2 Fig). We found two distinct populations (representing the two barcode orientations), with an average nucleotide difference of 7 bases. Genetic analysis also revealed 105 barcodes with one or more base pair deletions generating slightly smaller barcodes. These short inserts are likely due to errors in the molecular generation of the barcoded clone and although these barcodes are truncated, they retain their usefulness because they can still be genetically distinguished from the rest of the variant pool. Overall, these data support the conclusion that we have generated a genetically diverse, synthetic viral population approaching 10,000 individual viral clonotypes.

### SIVmac239M *in vitro* and *in vivo* infectivity and replication dynamics

**Fig 2:**
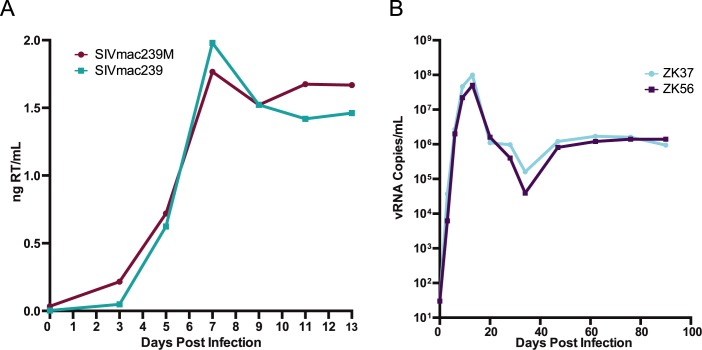
SIVmac239M *in vitro* and *in vivo* replication. (A) CD8+ T-cell-depleted PBMCs infected with either SIVmac239 (teal) or SIVmac239M (maroon) were monitored over 13 days and samples collected were assayed by ELISA specific for reverse transcriptase (RT) over time. (B) Infectivity was tested *in vivo* in two rhesus macaques, ZK37 (light blue) and ZK56 (purple) following intravenous infection. Viral RNA copies were measured in plasma over 100 days post infection with a lower limit of detection of 15 copies/mL.

Prior to use in nonhuman primates, viral infectivity and replication of SIVmac239M was assessed using TZM-bl reporter cells and primary rhesus lymphocytes, respectively. This stock contained 2.2x10^5^ IU/mL, which was equivalent to the infectious titer of a stock of parental SIVmac239 produced using the same approach. To assess the replication capacity of SIVmac239M, CD8+ T-cell-depleted PBMCs were inoculated with equivalent infectious units of SIVmac239M or the parental SIVmac239 and samples were collected every 2–3 days (Fig 2A). SIVmac239M displayed peak virus replication levels on day 7, corresponding to a detected 1.8ng of reverse transcriptase (RT)/mL of culture supernatant. The viral growth curves were comparable between SIVmac239M and parental SIVmac239, which also peaked on day 7 with 2.0ng of RT/mL. These results demonstrate that the insertion of the barcode into the viral genome did not have a measurable deleterious effect on either infectivity or replicative capacity *in vitro*.

To confirm that SIVmac239M did not have any replication defects, we assessed its replication capacity *in vivo* in rhesus macaques. Two rhesus macaques (ZK37 and ZK56) were infected with 2.2x10^5^ IU of SIVmac239M via intravenous injection. Plasma viral loads were monitored regularly using qRT-PCR. SIVmac239M displayed viral replication kinetics comparable to wild type SIVmac239 [27] resulting in peak viremia in both SIVmac239M infected animals at day 13 with viral RNA copies at 5.0x10^7^–1.0x10^8^ copies/mL (Fig 2B). Viral RNA set-point was reached by day 49 with titers at ~10^6^ copies/mL, which was similar to parental SIVmac239 [27]. These data indicate that SIVmac239M is fully functional *in vivo* with viral kinetics indistinguishable from wild-type SIVmac239 and suggest that the insertion of the barcode did not result in impaired infectivity or replicative function. Furthermore, sequence analysis through 3 months post infection revealed no loss of detected barcodes or accumulated changes that precluded barcode tracking and variant enumeration. Sequence analysis of plasma during chronic viremia revealed all viral genomes contained a barcoded insert. It was also observed that while mutations did occur, they were uncommon in the 34 bases of the barcode insert. On occasions when mutations did affect the barcoded region, the parental barcode was identifiable phylogenetically.

### Evaluation of barcodes during acute infection

The underlying design premise in using SIVmac239M for studies of viral reservoirs was to establish a disseminated systemic infection with a large number of sequence-discriminable viral variants that are isogenic outside of the barcode and biologically equivalent. As each different variant represents the progeny of a distinct chain of infection events, barcode sequence analysis in SIVmac239M infected animals undergoing viral recrudescence after cART discontinuation should allow for unprecedented facility and depth of analysis while limiting confounding diversification and differences in viral replication capacity or other biological properties that can accumulate over time in the infected host prior to initiation of cART. To achieve reservoir seeding with numerous barcode variants, we employed a relatively high dose intravenous infection, while allowing limited time for viral replication before initiation of cART to control the size of the reservoir. The design premise of this study was to mimic the diversity of viral variants capable of seeding persistent viral reservoirs in chronic HIV infection without the attendant biological variability. It was therefore necessary to determine whether SIVmac239M could establish a genetically diverse infection in rhesus macaques. To assess the number of detectable barcodes in plasma during primary infection, plasma from day 4 to day 14 post-infection was obtained from 26 animals infected with SIVmac239M (including ZK37 and ZK56). Sequence analysis revealed an average of 1,247 clonotypes per animal (244–4800). The number of barcodes identified for individual animals varied based on duration of infection prior to treatment. Of the 9,336 confirmed barcodes in the stock, 7,991 (85.6%) were identified in at least one animal. Furthermore, each barcode was identified in a mean of 5.2 out of 26 animals, with an interquartile range of 1–8 animals. Because the large majority of barcodes from the stock could also be detected in animals, we conclude that at least 85.6% of the barcodes in our total stock are both infectious and functional.

**Fig 3:**
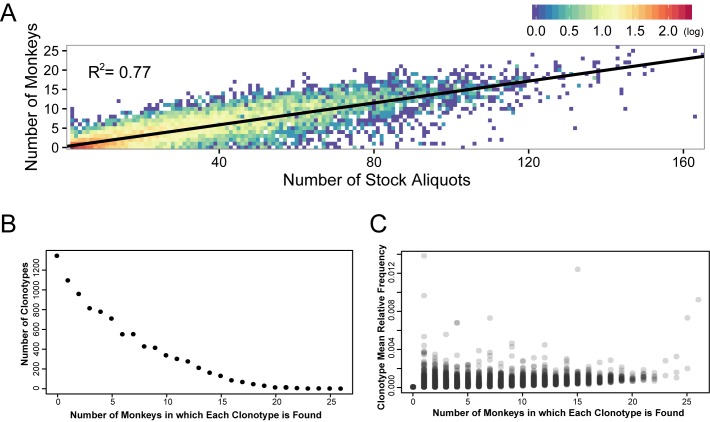
Evaluation of clonotypes found in stock and monkeys. (A) Each individual clonotype was plotted by its rank order in the stock versus the number of animals in which it was found. A linear correlation was generated with an R^2^ value of 0.77. Density of clonotypes at any single point are colored using a log scale heat map where red points depict 2 logs of clonotypes and dark blue points represent single clonotypes. (B) The number of barcodes was plotted against the number of monkeys in which the barcodes were found. Of the 9,336 total stock barcodes, 7,991 were found systemically in at least one animal, and 1,345 were not found in any of the 26 animals. (C) The mean relative frequency of each individual barcode (grey circles) was plotted against the number of monkeys in which the barcode was found. The relative frequencies of the barcodes demonstrate the comparative homogeneity of all clonotypes across all animals.

Because variation in the proportion of each clonotype within the stock was observed, the correlation between the frequency of the clonotype in the stock and frequency of detection of the clonotype during acute infection in animals was determined. Comparing the number of viral stock replicates in which a particular clonotype was identified (out of 168 total replicates) against the number of animals in which each clonotype was found yielded a linear correlation with an R^2^ value of 0.77 (p<10^−5^ Pearson/Spearman, Fig 3A). Thus, clonotypes that were identified frequently in the 168 replicates in the stock were also found in more animals after *in vivo* challenge. Importantly however, clonotype abundance in each animal (i.e., the relative number of copies of each clonotype) was only weakly correlated to their abundance in the viral stock (Spearman p-values ranging from 0.07–0.18), suggesting that although clonotypes found more frequently in the stock were more likely to infect an animal, they did not necessarily become the dominant clonotypes within the animal. These results highlight the biologically consistent nature of the different clonotypes and a lack of negative impact on viral fitness due to a barcode insertion. Further analysis of the distribution of barcodes in animals revealed an inverse relationship between the number of animals in which a barcode was found (i.e. animals sharing a common clonotype), and the number of common barcodes found in that number of animals (Fig 3B). That is, individual barcodes were found most frequently in only 1 animal, and least frequently in all 26 animals. The mean relative frequency of each clonotype was calculated by averaging the proportional abundance at which it was found in each individual animal. This value was then correlated with the number of animals in which that clonotype was identified. The number of animals in which each clonotype was found appears to be largely independent of its mean relative frequency in these animals, and each clonotype was found at nearly equal proportions in each animal with no single clonotype dominating the population. The fact that a barcode was found in many animals was therefore not due to greater fitness, as barcodes found in more animals did not represent larger proportion of the total sequences than barcodes observed in only a few animals. (Fig 3C). Thus, while the proportion of each clonotype in the stock correlates with the likelihood of establishing systemic infection, there is no indication that clonotypes differ in their replicative capacity.

### SIVmac239M short-term cART treatment and interruption

**Fig 4:**
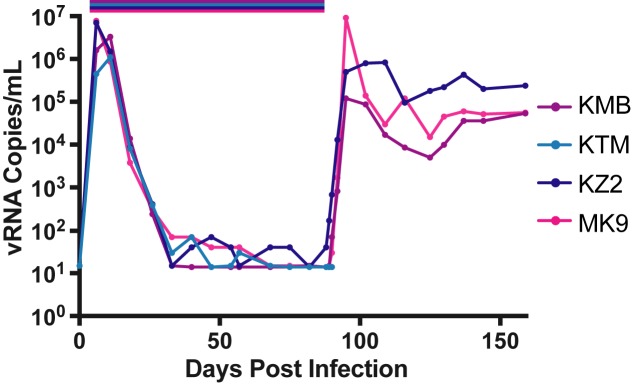
Plasma viral loads in animals in study 1. Four rhesus macaques, KMB (purple), MK9 (pink), KZ2 (navy), and KTM (blue) were infected with SIVmac239M on day 6 and treated with cART for 82 days (study 1). Viral RNA copies were measured in plasma over 150 days.

Our major goal in generating a barcoded virus was to facilitate the ability to discriminate between individual rebound events contributing to viremia following treatment interruption. A pilot study using short-duration cART treatment was initiated to test the feasibility of using the barcoded virus model system to discriminate distinct viral lineages following cART interruption (study 1). Here, 4 rhesus macaques (MK9, KMB, KZ2, and KTM) were each infected intravenously with 2.2x10^5^ IU of SIVmac239M followed by daily cART (TFV/FTC/RAL) administration from day 6 to day 88 post-infection, at which time cART was discontinued (Fig 4). Cell associated-viral RNA (CA-RNA) levels in PBMC peaked on day 6 post-infection at an average of 7.6x10^5^ copies/10^6^ cells (S3A Fig). CA-RNA levels dropped dramatically on cART to an average of 6.3 copies/million cells at time of interruption. CA-viral DNA (CA-DNA) also peaked on day 6 at an average of 2.0x10^4^ copies/10^6^ cells (S3B Fig). These DNA levels declined over the course of cART treatment, but more gradually than CA-RNA, reaching an average of 5.5x10^2^ copies/ 10^6^ cells at the time of interruption. The plasma SIV RNA viral load was below the assay quantification limit (15 copies/mL) from day 68 to treatment interruption for 3 of 4 animals (MK9, KMB, and KTM). Viral rebound was detectable in plasma 1–2 days following cART interruption, and plasma viral loads at rebound peak were between 1.2x10^5^–9.2x10^6^ copies/mL. Animal KZ2 never achieved full viral suppression, and had a detectable viral load (40 SIV RNA copies/mL) on the day therapy was interrupted, which rapidly increased thereafter, highlighting the lack of full suppression in this cohort. KTM died due to procedural complications at the time of cART interruption and was therefore excluded from subsequent analyses. These data reveal typical early replication dynamics, with a greater than 5-log reduction in plasma vRNA and CA-RNA during cART treatment and a less than 2-fold decrease in the CA-DNA levels during therapy. These animals displayed rapid rebound kinetics following cART interruption.

Sequence analysis just prior to initiation of therapy identified 1,872 distinct clonotypes in animal MK9, 1,815 in animal KMB, and 3,739 in animal KZ2. To assess the number of detectable rebounding clonotypes, next generation sequence analysis was performed 7 days post-interruption. In contrast to the pre-treatment viral diversity, following cART interruption, recrudescent rebound viremia contained only 118 distinct clonotypes for MK9, 136 for KMB and 87 for KZ2 (S4 Fig). Therefore, despite the limited duration of therapy, the number of detectable clonotypes in plasma was greatly reduced from the pre-therapy time point. For animal KZ2, which had detectable virus at the time of interruption, of the 87 detectable rebounding variants, one clonotype was found at 2 logs higher proportion than the next detectable clonotype. The relative proportion of all other clonotypes were within half a log of its nearest neighbor. Overall, these results indicate that individual clonotypes can be detected in plasma viremia immediately following cART interruption and, with sufficient template input, the relative proportion of each clonotype may be accurately assessed across 5 logs.

To confirm the reproducibility of the MiSeq sequencing to consistently provide proportional representation of virus populations and to determine if additional barcodes could be identified with a larger template input, barcode sequencing of rebound plasma viremia was repeated for MK9 but with a starting template input 10-fold higher than first assayed (total 1x10^6^ vRNA template copies assessed). Upon comparison of the relative abundances of sequenced clonotypes, nearly identical proportions of detected clonotypes were observed (S5 Fig). Furthermore, 32 additional barcodes were identified below the previous lower-limit of detection of 0.001%. These results confirm the reproducibility of our sequencing approach and that template input quantity determines the lower limit of detection.

### SIVmac239M long-term cART treatment and interruption

**Fig 5:**
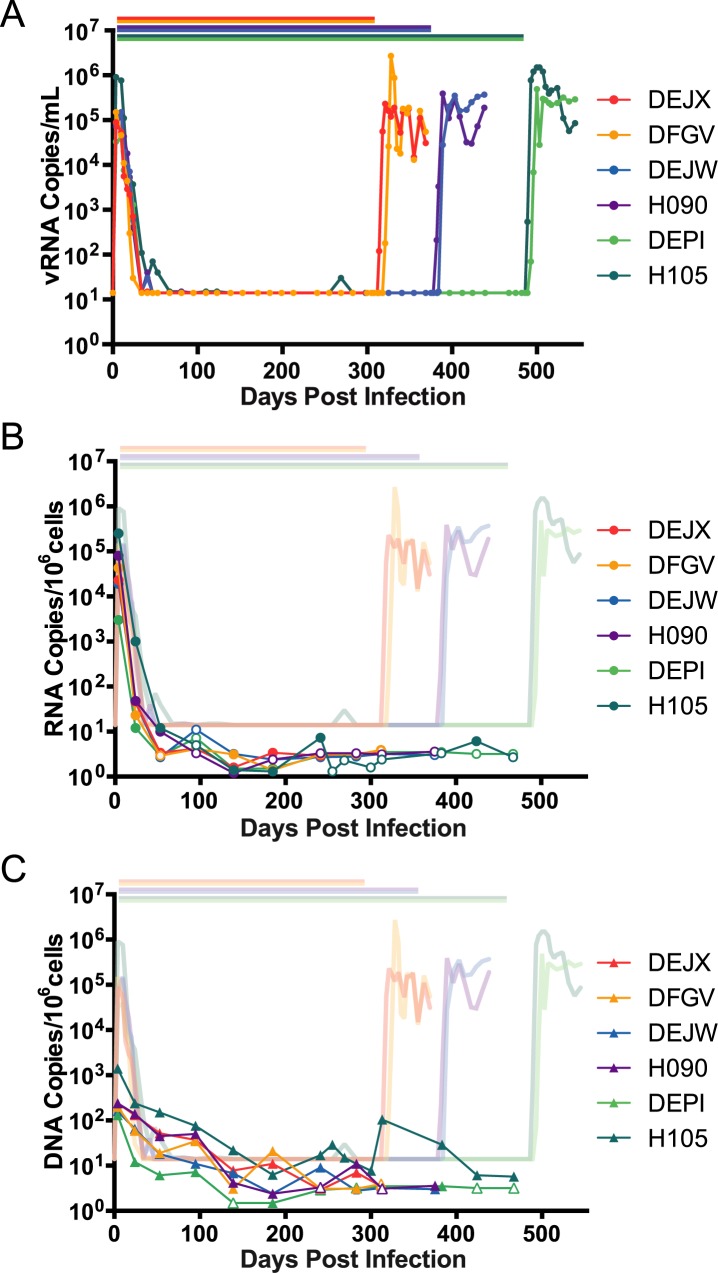
Plasma viral loads, PBMC CA-vRNA and CA-vDNA in animals in study 2. (A) 6 rhesus macaques, DEJX (red), DFGV (yellow), DEJW (blue), H090 (purple), DEPI (light green), and H105 (dark green) were infected with SIVmac239M and cART was initiated on day 4 post-infection. Pairs of macaques were removed from therapy on days 305, 374, and 482 post-infection. Viral RNA copies were measured in plasma collected up to 550 days. Bars over the viral load data indicate duration of therapy for each animal. (B-C) Cell-associated viral DNA (B) and RNA (C) was measured in PBMCs over the duration of cART therapy in animals infected with SIVmac239M. Measurements obtained are shown overlaying the detected plasma viral loads. Open symbols represent measurements that were below the limit of detection.

This short-term cART treatment study demonstrated that SIVmac239M could be used to enumerate transmitted/founder variants before cART and the rebound/founder variants following cART interruption. However, 82 days of therapy was insufficient for full suppression and was likely not long enough for the decay of all short-lived viral reservoirs, therefore, a longer-term cART suppression study was conducted (study 2). Six rhesus macaques infected intravenously with SIVmac239M were given cART (TFV/FTC/RAL/IDV/RTV) starting on day 4 post-infection. Viral load measurements revealed rapid acute phase kinetics with viral load measurements ranging from 3.3x10^4^–9.1x10^5^ copies/mL at day 4 post-inoculation. Plasma viremia decreased over the next 9 weeks, and by day 67, all animals were suppressed to below 15 copies/mL (Fig 5A). Plasma viral loads were maintained below 15 copies/mL apart from one viral blip (30 copies/mL) in animal H105 at day 269 post-infection, which happened to coincide temporally with a diagnosis of dermatitis and associated topical antibiotic treatment.

At day 305 post-infection, therapy was discontinued in 2 animals (DEJX and DFGV). Viremia was first detected at day 9 post-cART for DEJX and day 16 for DFGV. Peak post-cART viral loads of 2.3x10^5^copies/mL for DEJX and 2.7x10^6^ copies/mL for DFGV were measured at day 16 and day 23, respectively. At day 374 post-infection, therapy was discontinued in an additional 2 animals (H090 and DEJW). Viremia was detected at days 7 and 9 post-cART with peak viral loads of 3.9x10^5^ copies/mL on day 15, and 3.5x10^5^ copies/mL on day 29 for H090 and DEJW, respectively. The final two animals (H105 and DEPI) discontinued treatment on day 482. Viremia was first detected on days 7 and 11 with peak rebound viral loads of 1.5x10^6^ copies/mL on day 18 for H105, and 4.9x10^5^ copies/mL on day 18 for DEPI. In this study, the time to detectable viremia ranged from 7 to 16 days post-cART, which was significantly longer than animals in study 1 (p = 0.004, Log-rank Mantel-Cox test). Notably, the time to rebound is much faster in these studies than has been reported for human studies [28]. However, there was no significant difference in peak rebound viremia between study 1 (mean 3.4x10^6^ copies/mL) and study 2 (mean 9.4x10^5^ copies/mL) (p = 0.26).

CA-RNA and DNA were isolated from PBMCs collected regularly over the course of the study and quantified using real-time PCR/RT-PCR. For animals in study 2, CA-RNA levels peaked at 3.0x10^3^–2.5x10^5^ copies/10^6^ cells on day 4 (immediately prior to the initiation of therapy) (Fig 5B). CA-RNA fell below 10 copies/10^6^ cells by day 53 in all animals and remained at or below the limit of quantification until interruption from cART. CA-DNA levels peaked at 1.3x10^2^–1.4x10^3^ copies/10^6^ cells immediately prior to the initiation of suppressive therapy which diminished more slowly than CA-RNA levels, but reaching 10 copies/10^6^ cells by day 283 (Fig 5C). Animal H105 showed a spike in CA-DNA on day 313, but was again near the limit of quantification by day 430 and remained suppressed for the duration of therapy. This CA-DNA spike was preceded by a blip in plasma viral load at day 269. No corresponding increase in CA-RNA was observed. The peak levels of CA-DNA and CA-RNA at cART initiation and the levels at interruption were markedly higher in animals in study 1 where treatment began on day 6 and lasted for only 82 days compared to those in study 2 in which treatment began on day 4 and lasted for more than 300 days (p<0.001, t-test). These differences in viral DNA and RNA levels highlight the importance of the dynamics of acute infection and timing of treatment initiation on the establishment of the viral reservoir (19, 37) and provide a means to control the reservoir size based on time to cART initiation.

**Fig 6:**
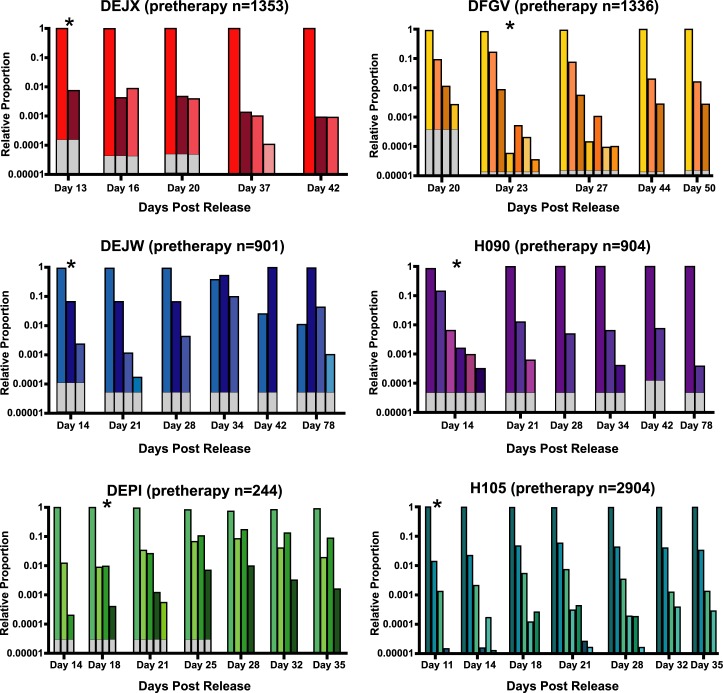
Longitudinal rebound clonotypes. The relative proportion of the clonotypes in the six animals in study 2 were identified at multiple time points following therapeutic interruption. Each color represents a unique barcode. Although individual barcode numbers are not shown for simplicity, no rebound clonotypes were found in more than one animal. White portions of each column denote the area below the limit of detection for each sample based on template input quantities. Time points denoted with an asterisk indicate the time-point corresponding to the highest viral titers in the exponential growth phase.

Sequence analysis of the clonotypes detected in plasma prior to cART treatment in study 2 revealed patterns similar to those seen prior to therapy in study 1. The average number of detectable barcodes at day 4 (peak pre-therapy) was 1,274. Post-rebound sequencing was performed on samples obtained from ramp-up to early set-point viremia to identify the number of rebounding clonotypes and assess their relative abundance over time (Fig 6). Across all six animals, we detected a total of 34 unique rebounding clonotypes ranging from 4–8 variants per animal (overall mean of 5.7), with a maximum of 7 clonotypes detected at any given time, with no clear difference in animals that discontinued therapy on day 305 (mean of 5.5 clonotypes), day 374 (mean of 5.0 clonotypes), or day 482 (mean of 6.5 clonotypes). For animals DEJX, DFGV, H090, DEPI, and H105, the proportions of variants remained substantially consistent over time in the initial weeks after cART discontinuation, with only the minor clonotypes showing any notable variation. Interestingly, for animal DEJW, the dominant clonotype during rebound (clone 4886) was replaced at day 35 post interruption by the second most dominant clonotype (clone 997). Overall, these data are consistent with previous work showing a stable viral population over time with limited changes in variant proportion once those proportions are established [27].

Next, each rebounding clonotype was compared to its relative proportion in the same animal prior to cART initiation and to the SIVmac239M stock (S6 Fig). Of the 34 total detected rebound clonotypes, 27 were identified in the pre-therapy sample from the same animal. We considered whether some clonotypes may be more fit than others, despite their inherent clonality. While this was not directly measurable, it was noted that if some clonotypes were more fit, they would presumably emerge as rebounders in most or all animals used in this study. In fact, in study 2, no rebounding clonotype was observed in more than one animal, and even in study 1, in which we counted an average of 114 rebounding clonotypes per animal, less than 25% were identified in more than one animal, and only 1 was found in all three. Additionally, in both study 1 and 2, the clonotypes most abundantly represented prior to therapy were more likely to be detected following cART interruption, presumably because they were more likely to seed a larger number of cells capable of harboring stable residual virus (S7 Fig). We find significant correlation between the pre-therapy and post-rebound clonotypes in study 1 (p<0.001), but no significance in study 2, likely due to the limited number of rebounding genomes in this study. These observations are key as they demonstrate that it is possible to enumerate and identify clonotypes both before and after ART treatment. The observed clonotypes following ART interruption represent progeny from the activation of a long-lived viral reservoir.

### Estimating the frequency of reactivation after treatment interruption

A major goal in generating a barcoded virus was to establish a model in which both the number of sources of recrudescent virus and the dynamics of each rebounding variant could be directly estimated from the number and relative proportion of clonotypes detected in plasma. We propose that each reactivation event leading to systemic viremia can be identified using the relative proportion of each rebounding variant and the overall slope of the rebounding viral load curve. Assuming equivalent replication of each clonotype, the differences in the relative proportion of each detectable barcode can be used to infer the rate of reactivation from latency for each animal. The dynamics of viral recrudescence leading to measurable plasma viremia and the relative abundance of each clonotype sequence detected in plasma during acute recrudescence (time point highlighted by asterisk in Fig 6) were used to calculate the estimated frequency of reactivation from latency (or reactivation rate) (Eq 8). In animals in study 2 treated beginning at day 4 post infection for 305, 374, or 482 days, the estimated reactivation rates per day averaged 0.64, 0.59, and 0.41 respectively with an overall average of 0.54 –that is roughly one reactivation event every 2 days. In study 1, while the calculated reactivation rate was 16–31 events per day (mean 22.7, p = 0.02, Mann-Whitney Test), these rates likely reflect the residual presence of virus that was actively replicating or being actively produced during the relatively brief duration of cART prior to discontinuation, and thus may not represent reactivation from long-lived, latent cells. This interpretation is supported by the detectable viral load values for animal KZ2 prior to interruption from cART. Therefore, we cannot distinguish between plasma virus representing reactivating latently infected cells and residual viremia that, upon drug levels reaching a minimum threshold concentration, was immediately available to initiate a spreading infection.

**Fig 7:**
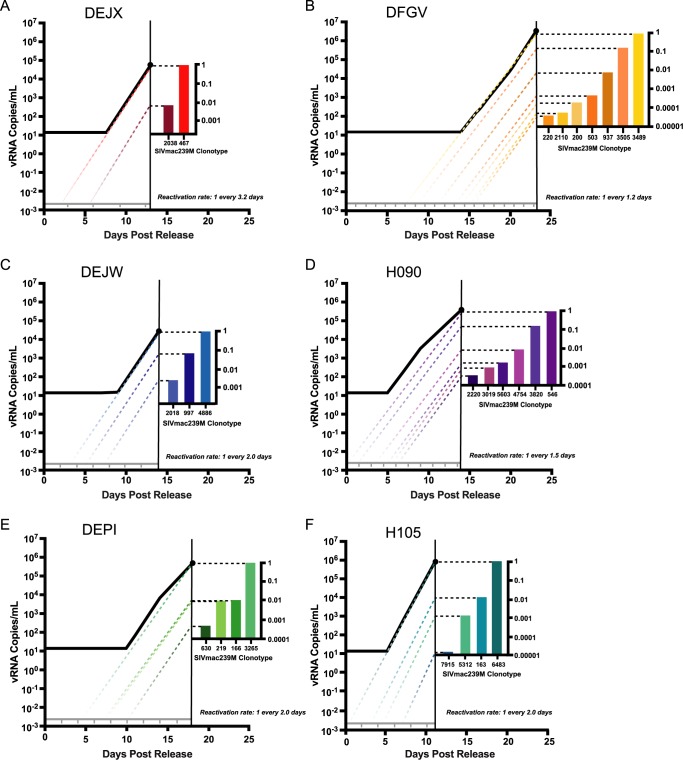
Pairing of relative viral loads and time of reactivation. Approximate day of reactivation of cells harboring clonotypes was estimated in study 2 animals following interruption of long-duration therapy for animals DEJX (A), DFGV (B), DEJW (C), H090 (D), DEPI (E), and H105 (F). Total viral load at the time point selected is comprised of the relative proportions of clonotypes determined by sequencing analysis. Each individual clonotype’s growth rate was estimated during the maximum exponential phase of the total viral load curve, and the slope of the growth of each clonotype was extended below the limit of detection to estimate the approximate time of reactivation. The grey line represents the plasma concentration of one viral copy in the total plasma, and the tick marks represent the theoretical reactivations based on calculated reactivation rate for each animal.

Using the proportion of the total viral load represented by each clonotype, and the exponential growth rate in plasma viremia, we extrapolated the slope of the viral load curve of each animal from study 2 to below the limit of detection for each detected clonotype to the theoretical concentration of a single virion in the total blood volume, calculated using a standard clinical volume of plasma in a rhesus macaque (54mL plasma/kg body weight) which equates to 2.6x10^-3^ viral RNA copies/mL in a typical 7kg animal (Fig 7). The reactivation rate was plotted across the x-axis, starting at time 0 post interruption from cART, indicating the estimated average time interval between each reactivation event within each animal. Although reactivation of a latent provirus in an individual cell is stochastic in nature, we found most reactivations occurred within the predicted window of time (i.e., within the time-frame predicted by the calculated reactivation rate). In 3 animals, we hypothesized that a theoretical reactivation event occurred within the first window of reactivation (DEJX, H090, and H105), and posited that reactivation occurred shortly after therapy was discontinued with limited time for drug washout. For animal DEJW, an activation event likely did not occur within the first predicted reactivation window, but afterwards all three detectable clonotypes fit within the inferred reactivation windows. For animal DEPI, a reactivation event did not occur in the first 2 reactivation windows, but did for subsequent windows. The time to rebound in animal DFGV was the most delayed and we estimate that the first 6 reactivation windows were missed prior to a robust reactivation of many viral lineages. The theoretical initial reactivation events occurred in animals DEJX, DFGV, DEJW, H090, DEPI, and H105 on days 1.8, 7.3, 3.2, 0.5, 4.3, and 0.3 days after treatment interruption.

## Discussion

Accurately assessing the size and nature of the residual viral reservoir that can give rise to recrudescent viremia is essential for studies focused on prolonging cART-free remission. In addition to various *ex vivo* assays, all of which have significant limitations, many current assessments of reservoir-targeting therapeutic strategies include *in vivo* testing in HIV+ individuals, followed by cART interruption and monitoring of time to detectable viremia. However, treatment interruption likely causes reseeding of the viral reservoir during viral recrudescence off therapy, regardless of how short a time interval the interruption lasts, and the potential consequences of this must be carefully considered. Using treatment interruption and time-to-detection of virus to measure changes in the frequency of reactivation and then extrapolating this frequency to estimate changes in reservoir size after reservoir-targeting therapeutic strategies has low statistical power to detect the effects of therapy [17]. Moreover, if reactivation rates are high, or highly variable, then this will further reduce the probability of successfully assessing new interventions [29]. In the absence of any identified strategies that robustly diminish the size of the residual virus pool on cART by multiple logs, it will be important to develop approaches that allow for the identification of those therapies that have a more modest effect on the size of the viral reservoir but that may be improved upon or useful in combination with other agents. Nonhuman primates offer useful models for the assessment of intervention strategies as reservoirs can be established to recapitulate those established in HIV-1 infected patients, and interruption can be studied without risk to patients. For studies of reservoir targeting strategies, it is advantageous for the size of the viral reservoir to be controlled and normalized between experimental groups. This kind of experimental stringency is possible in NHP models in a way that is impossible in humans. Here we demonstrate a barcoded virus model to assess reservoir size which, when taken together with time to rebound measurements, provides a more sensitive and robust assessment of therapeutic changes to the latent reservoir.

Viral RNA collected from human plasma following rebound has been sequenced to estimate the number of latent cells reactivating [17], as the diverse viral quasi-species in chronic HIV infection allows for enumeration and characterization of viral reservoirs and recrudescence. While this previous work has shown that multiple viral lineages can contribute to rebounding viremia (28–31), they are limited in the depth of sequencing, the small region of viral sequence analyzed, and limited overall viral diversity in HIV infection to distinguish individual reactivation events. One of the possible problems with attempting to use this method with human samples is that sequencing of *env* or *pol* requires SGA to evaluate the genetic diversity of each sample [30–32], analysis that is labor intensive and can feasibly only yield a maximum of approximately 100 sequences. Analysis of 100 sequences from a patient with viral loads greater than 10^4^ copies/mL will only allow for the detection of lineages representing the largest proportions of that population. An alternative approach would be to preselect individuals with greater overall viral heterogeneity and use a diverse region of the genome to identify the relative proportion of variants [17, 33]. While the relative proportions of each lineage can still be estimated with these approaches, utilizing current technology, they lack sufficient depth and dynamic range to accurately assess reactivation rates in most HIV-1 infected individuals. By contrast, our clonal barcoded virus is genetically homogeneous, with only the short stretch of 34 bases harboring the entire genetic disparity between clonotypes. Because only this short portion of the genome requires analysis to distinguish between lineages, we are able to use next generation sequencing, a method that reads tens of thousands of individual sequences, allowing for detection of rare clonotypes and consistent evaluation of the relative proportions of rebounding lineages. When evaluated in this manner, the proportion of clonotypes is reflective of the timing of the individual reactivation events that led to each viral lineage. This measured rate may therefore be used to assess the successful intervention of novel therapeutics that can reduce the overall viral reservoir size. This type of evaluation is entirely dependent on the assumption that each individual clonotype replicates at an identical rate, emphasizing the utility of our clonal virus model, and further highlights the complication of utilizing this approach in HIV-1 infected patients. Admittedly, despite the clonal nature of SIVmac239M, one factor potentially complicating analyses based on both time to detection and reactivation rates is the anatomic location of each rebounding lineage. Reactivation at sites with a limited number of target cells available for rapid viral expansion might alter the inferred reactivation rate. However, this error is minimized when using reactivation rates as a measure of reservoir size because the rates are based on an average of multiple reactivation events and variation in growth rate of a few rebounding clonotypes will not alter the calculated rate. By contrast, if using only time to detection as an estimate of reservoir size, the variation in target cell availability would significantly impact the time to viral detection, because this assessment is based only on a single measurement (i.e., the time needed for the first reactivating latent reservoir to produce detectable virus).

In order for a barcoded virus to allow the evaluation of the latent reservoir, each viral variant must be functionally equivalent. With only a 34-nucleotide cassette inserted between the *vpx* and *vpr* genes, there is no apparent effect on *in vitro* or *in vivo* infectivity or replication capacity of the virus. Of 9,336 distinct, apparently biologically equivalent clonotypes present in the stock, 105 clonotypes were found to bear truncated barcode inserts, representing 1.1% of the total stock population. Hypothesizing that these smaller barcodes might confer some fitness advantage compared to clonotypes with the full 34 base insertion and that resultant viral populations might show bias for these clonotypes, we examined all pre- and post-therapy populations to determine the relative contribution of these clonotypes to the total viral load. We found that these barcodes do not overwhelm the pre-therapy population nor do they prevent full length clonotypes from also establishing infection and reservoirs. Even the presence of wild-type SIVmac239 and the SIVmac239 with only the MluI restriction site but no barcode did not cause a disproportionate bias in the population. This emphasizes the fact that the insertion of the genetic cassette has no discernable negative effects on successful replication of the virus. Additionally, stochastic mutations elsewhere in the genome could potentially allow for selection, however in this study, animals were placed on suppressive therapy on days 4 and 6, which limited the time for a fitness-conferring mutation to arise, and therefore upon therapy release, no genome was advantageously poised to dominate the population.

One of the most common methods for evaluating the relative size of the latent reservoir in HIV+ individuals is through the quantification of CA-DNA in PBMCs. In our study, early treatment appears to have constrained the level of CA-DNA in the animals, with all of the animals receiving long-term therapy in study 2 having fewer than 10 copies of CA-DNA per million cells by day 53 post-infection, and even the animals receiving short-term therapy in study 1 reaching minimum levels of only 387–650 CA-DNA copies per million cells. These low levels unfortunately prevented the collection of a meaningful number of CA-DNA and RNA sequences to compare with rebounding clonotypes in the plasma. More extensive sampling and analysis in future studies may allow assessment of potential correlations between various estimates of reservoir size based on *ex vivo* assays and based on the method presented here. Early treatment also likely prevented elevated levels of immune activation/inflammation that persist in cART suppressed individuals and animals initiating cART during chronic infection [34, 35].

One of the major advantages to using NHP models for reservoir research is the ability to manipulate reservoir size and the resultant number of rebounding variants following treatment interruption by controlling standardized inocula, timing of cART initiation, and duration of treatment. In our study, the animals that received cART beginning on day 6 post-infection for 82 days (study 1) had approximately 10 times higher frequency of rebounders than animals started on cART beginning on day 4 for 305–482 days (study 2). There may be two explanations for this. First, these animals were treated on day 6, two days later than the day 4 treated animals in study 2, therefore viral seeding, which expands logarithmically during the acute (pre-peak viremia) phase of infection, reached higher levels prior to suppression. This hypothesis is supported by the fact that the CA-DNA levels just prior to cART initiation and just prior to cART discontinuation are approximately 2 logs higher in the animals in study 1 (averaging approximately 10^3^ copies/10^6^ cells), compared to the animals in study 2 (averaging approximately 10 copies/10^6^ cells). Because the seeded latent reservoir is larger, this could explain why both the rate of reactivation and the number of rebounding clonotypes is significantly higher (p = 0.01, Mann-Whitney Test). An additional factor potentially contributing to the increased number of detectable rebounders is that the duration of suppressive therapy was much shorter in the animals in study 1, and it is likely that cells actively producing replication competent virus have not had sufficient time to decay down to minimal quantities. This would dramatically increase the apparent size of the reservoir, and result in a much greater rate/number of reactivating cells. Additionally, full viral suppression was likely not achieved in these animals, as evidenced by the detectable viral load of KZ2 on the day of therapy interruption. This suggests the presence of residual production of virus in the animal which, upon degradation/metabolization of cART, could potentially initiate recrudescent infection, given susceptible target cells. As such, there is no way to distinguish between those clonotypes that arose from spread of variants present as residual viremia and those that resulted from activation of latently infected cells. These confounding factors likely contribute to rebound viremia in the animals in study 1, whereas by the time of cART interruption in the animals in study 2, these shorter-lived reservoirs likely had decayed or been eliminated, and residual viremia cleared from the system. Furthermore, animals DEJX, H090, and H105 were also detectable quickly within the first few days, suggesting drug washout is rapid in these animals. Interestingly DFGV had a similar rate of reactivation to the other animals in study 2, but had a considerable delay in time to first detectable viremia likely due to variability in drug washout kinetics. This animal to animal variation might be important for studies that utilize only time to detection as the measure of viral reservoir size.

To estimate the decrease in reactivation rate in animals in study 2, a decay curve was fitted to our data (*y* = *y*_0_*e*^*kt*^, where *y* is the reactivation rate, and *t* is the duration of treatment). We find that time required for the reactivation frequency to decrease by 50% is 299 days. Ultimately, it is most likely that both peak viral load and duration of therapy account for the differences in the number of rebound variants we observe in the two studies. This then illustrates a major advantage of an animal infection model paired with our barcoded virus, namely, that we may control the timing of cART initiation and treatment duration to modulate reservoir size to fit the desired parameters of the study. Most animal studies conducted to measure reservoir size allow infection to reach chronic phase prior to initiation of therapy, and then utilize time to detection of viral load to identify changes in reservoir size. Time to detection is a valid method for monitoring changes in reservoir size, however it requires large sample group sizes to detect the effects of reservoir reduction [17, 29]. Our model adds power and depth to the traditional time to detection measurement. By using a barcoded virus and next generation sequencing, we greatly increase the sensitivity of detecting changes in the reservoir size by increasing the amount of information that can be derived from each animal. The measured number of rebounding clonotypes and the corresponding reactivation rates are reflective of the functional reservoir size, and thus may be used as a direct measurement of the latent reservoir that effectively contributes to rebound viremia.

When this study was initiated, it was theorized that early treatment itself could limit the viral reservoir and prevent rebounding viremia [36]. One sobering observation made here was that even after very early treatment (day 4 post-infection) and over a year of suppressive therapy, viremia rapidly recrudesced after treatment interruption. Despite the early treatment start date, a time frame which is virtually impossible to achieve for newly infected humans, we still detected viral rebound within eleven days of treatment interruption. Although delayed when treated early, previous studies in both humans [28, 37] and NHPs [19] demonstrate similar findings: that once viremia is detectable, despite early treatment, viral reservoir is irreversibly established and causes recrudescent viremia. These studies and our findings highlight the urgency of developing novel therapeutics to target the reservoir directly. Furthermore, it remains to be determined if post-rebound control of viremia can be augmented by some intervention strategy, thereby providing a functional cure if elimination of viral reservoirs cannot be obtained.

There are likely numerous applications for a barcoded virus. This would be an ideal system for sensitive detection of minor changes in reservoir size induced by latency reversing agents or other adjunctive therapies. Additionally, this barcode could be introduced into other lentiviruses used in nonhuman primate research, including SHIV clones and minimally chimeric HIV. It may also be useful if introduced into HIV-1 clones for *in vitro* testing and in humanized mice. This approach is also not limited to lentiviruses, and might be useful for other viruses that would tolerate a small genetic barcode. Furthermore, this approach might extend to other replicating biological systems that could benefit from genetic tracking, including bacteria and fungi. A major advantage of the model is that because the genetic insert is small, it reduces the probability of exerting any inhibitory effect on growth or infectivity and is it unlikely to be extruded from the genome.

## Supporting information

S1 Fig Phylogenetic tree of barcoded stock. Neighbor joining phylogenetic tree depicting each individual barcode in the stock (n = 9,336). Branches shown in magenta represent barcodes inserted into the SIVmac239 backbone in one direction, and those shown in teal represent those inserted in the opposite direction.
(EPS)

S2 Fig Genetic distances between barcodes. The histogram depicts the distribution of the number of nucleotide differences between each pair of barcode sequences (n = 43,575,780 pairs).
(EPS)

S3 Fig PBMC CA-vRNA and CA-vDNA in animals in study 1. Cell-associated viral RNA (A) and DNA (B) were measured from PBMCs. Measurements obtained are shown overlaying the detected plasma viral loads.
(EPS)

S4 Fig Identification of rebound clonotypes after therapy interruption in study 1 animals. Plasma vRNA was collected from animals MK9, KMB, and KZ2 and analyzed following deep-sequencing to identify the barcodes present on day 7 post-ATI. 118 clonotypes were identified in MK9, 136 were identified in KMB, and 87 were identified in KZ2. The identified clonotypes are shown based on their relative abundance in each animal.
(EPS)

S5 Fig Comparison of template input values on the number of clonotypes detected during viral rebound. For animal MK9, sequence analysis of 10^5^ (green) and 10^6^ (blue) input templates were compared. Identified clonotypes are plotted based on their measured relative proportion, and matched with the corresponding clonotype in the other input sample. While the vast majority of all clonotypes were found in both duplicate assays and at nearly the same relative proportion, additional template input allowed for the identification of supplementary barcodes.
(EPS)

S6 Fig Pre-therapy clonotypes identified in study 2 animals. (A-F) The clonotypes present at peak viremia in study 2 animals prior to treatment were identified. Arrows indicate clonotypes that were identified following interruption of suppressive therapy. Arrows shown off the graph represent rebound barcodes that were not identified before initiation of therapy. (G) For each individual clonotype, the number of monkeys versus the number of aliquots of the stock in which it was found is plotted (greyscale of Fig 4A). The rebounding clonotypes are superimposed on the plot where the color of each dot corresponds to a clonotype from the indicated monkey.
(EPS)

S7 Fig Relationship between rank of rebound clonotypes with rank before treatment. Clonotypes identified in animals before treatment (at peak viral-load) were plotted against the rank order of the rebound clonotypes at day 7 post-ATI (study 1), or at the time point corresponding to the highest viral load during exponential viral growth post-ATI (study 2).
(EPS)
